# A Non-Volatile Tunable Terahertz Metamaterial Absorber Using Graphene Floating Gate

**DOI:** 10.3390/mi12030333

**Published:** 2021-03-21

**Authors:** Jinjun Bai, Wei Shen, Jia Shi, Wei Xu, Shusheng Zhang, Shengjiang Chang

**Affiliations:** 1 Tianjin Key Laboratory of Optoelectronic Detection Technology and Systems, School of Electrical and Electronic Engineering, Tiangong University, Tianjin 300387, China; wsshenwei@126.com (W.S.); shijia@tiangong.edu.cn (J.S.); xuwei@tiangong.edu.cn (W.X.); damutou2016@163.com (S.Z.); 2 Institute of Modern Optics, Nankai University, Tianjin 300071, China; sjchang@nankai.edu.cn

**Keywords:** floating gate, graphene, non-volatile, terahertz, tunable

## Abstract

Based on the graphene floating gate, a tunable terahertz metamaterial absorber is proposed. Compared with the traditional graphene–dielectric–metal absorber, our absorber has the property of being non-volatile and capacity for anti-interference. Using the finite element method, the paper investigates the absorption spectra, the electric field energy distribution, the tunability and the physical mechanism. In addition, we also analyse the influence of geometry, polarization and incident angles on the absorption. Simulation results show that the bandwidth of the absorption above 90% can reach up to 2.597 THz at the center frequency of 3.970 THz, and the maximum absorption can be tuned continuously from 14.405% to 99.864% by controlling the Fermi level from 0 eV to 0.8 eV. Meanwhile, the proposed absorber has the advantages of polarization insensitivity and a wide angle, and has potential applications in imaging, sensing and photoelectric detection.

## 1. Introduction

Terahertz (THz) waves have proven to be promising applications in the areas of imaging [1], communication [2,3] and biomedical science [4,5], due to their superior properties. However, the lag of THz devices has become one of the main obstacles to their application in practice, because of the lack of ideal functional materials. The emergence of metamaterial provides a way for the design and fabrication of THz devices. In particular, since it was reported in 2008 [6], the THz metamaterial absorber (MMA) has been attracting a lot of interest. The single-band [7,8,9], multi-band [10,11,12] and broadband [13,14,15] absorbers have been widely investigated. However, these absorbers cannot meet the tunable requirement in complicated electromagnetic circumstances.

Graphene possesses many unique electronic and optical properties, which can be applied in solar cells [16] and other photonic applications. The optical conductivity of graphene can be changed by electrostatic or chemical doping, so it can be used in tunable MMAs. Currently, several methods are available to design tunable absorbers, such as temperature [17,18,19], voltage [20,21,22,23,24,25], light [26,27] and mechanics [28]. Among them, the voltage tunable THz graphene MMA is dominant because it is convenient and flexible. There are two kinds of graphene tunable MMAs: frequency modulators [29,30,31,32,33,34] and amplitude modulators [35,36,37]. The former mainly include single-band and multi-band absorbers. The latter mainly include broadband absorbers. However, these absorbers are susceptible to interference, because their absorption behaviors are closely related with the external bias voltage.

To improve the anti-interference ability of the absorbers, we designed a non-volatile tunable THz MMA by using the graphene floating gate. In the off condition of external bias voltage, the absorption of the absorber can still maintain stability. Compared with the traditional tunable terahertz absorber, our absorber has the property of being non-volatile and capacity for anti-interference. Furthermore, the results indicate that the frequency range of absorption above 90% is 2.671 THz to 5.268 THz, and the tuning depth of maximum absorption is 85.575%. Meanwhile, the proposed absorber has the advantages of polarization insensitivity and a wide angle.

## 2. Structure and Design

**Figure 1:**
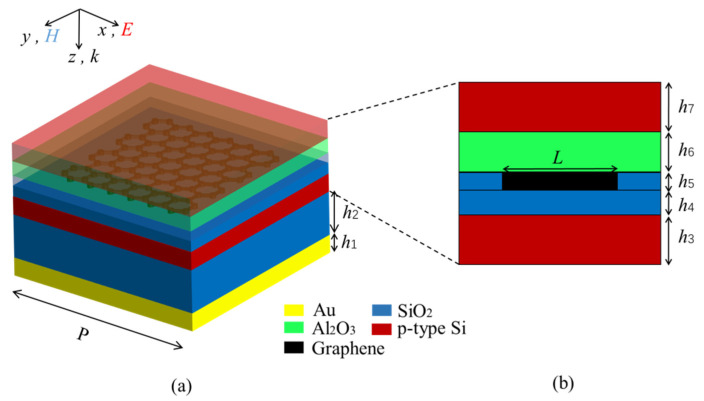
3D schematic (**a**) and side view of floating gate (**b**). The geometrical dimensions are *P* = 13 µm, *L* = 7.2 µm, *h*_1_ = 0.2 µm, *h*_2_ = 8.4 µm, *h*_3_ = 0.2 µm, *h*_4_ = 10 nm, *h*_5_ = 1 nm, *h*_6_ = 20 nm, *h*_7_ = 0.2 µm. The values of *h*_4_ and *h*_6_ are derived from reference [41].

**Figure 2:**
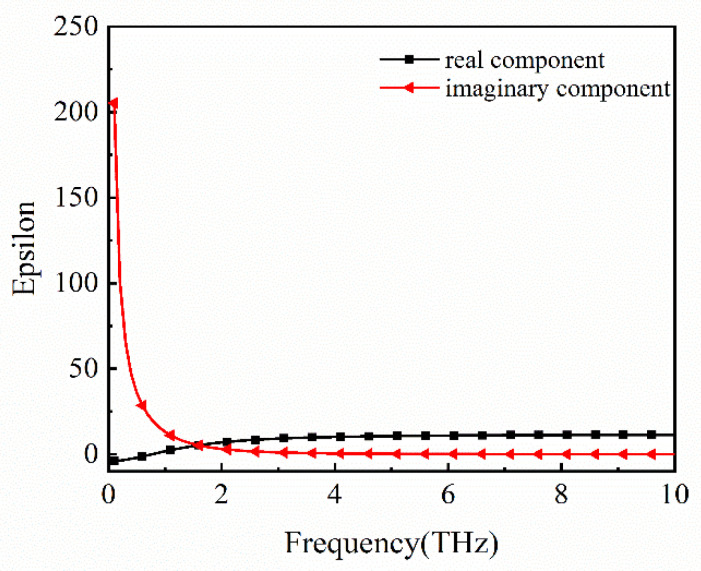
Dielectric property of the p-type doped silicon in THz band. It is described by the Drude model [14] as $\varepsilon =\varepsilon_{\infty }-\frac{\omega_{p}{}^{2}}{\omega^{2}+i\gamma \omega }$, where $\varepsilon_{\infty }=11.7$ is the constant permittivity at the infinite frequency, plasma frequency *ω_p_* and collision frequency *γ* are set to 2*π* × 5.22 THz and 2*π* × 1.32 THz, the corresponding carrier concentration and electron mobility are 8.8 × 10^16^ cm^−3^ and 814.3 cm^2^V^−1^s^−1^, respectively.

Figure 1a shows the periodic unit schematic of the proposed THz MMA, which has a square cross section. From bottom to top, the structure is composed of a substrate layer (gold), dielectric layer (silicon dioxide), lower conductive layer (p-type doped silicon), gate layer (silicon dioxide), graphene composite layer (graphene–SiO_2_), isolation layer (aluminum oxide) and upper conductive layer (p-type Si), where the graphene composite layer is made up of silicon dioxide embedded in a graphene block. The conductivity of gold is *σ* = 4.09 × 10^7^ S/m [38]. The dispersion properties are ignored. The permittivities of SiO_2_ and Al_2_O_3_ are *ε* = 3.80 [18] and *ε* = 9.39 [39], respectively. The dielectric property of the p-type doped silicon is shown in Figure 2. The transmission coefficient is equal to zero for the existence of a gold substrate. Therefore, the formula of absorption can be simplified as [40]: *A*(*ω*) = 1 − *R*(*ω*) = 1 − |*S*_11_|^2^, where *R*(*ω*) is reflectivity, S_11_ is reflection coefficient.

The graphene floating gate consists of the top five layers of the absorber, as shown in Figure 1b. It works like this [41]: with forward bias voltage (the upper conductive layer takes the positive power supply, the lower conductive layer is connected with the negative), the electrons in the lower conductive layer can tunnel through the gate layer into the graphene composite layer to increase its carrier concentration. Conversely, when a reverse bias voltage is applied, the electrons in graphene can tunnel through the gate layer, leading to a decrease in its carrier concentration. Note that the electrons cannot tunnel through the isolation layer because its thickness is twice that of the gate layer. When the bias voltage is disconnected, the graphene is isolated from the external environment so that its carrier concentration and the absorption of the absorber can remain unchanged. The non-volatile property of floating gates enhances the ability of the absorber of resistance to disturbance.

## 3. Results and Discussion

### 3.1. Simulation Results and Mechanism Analysis

**Figure 3:**
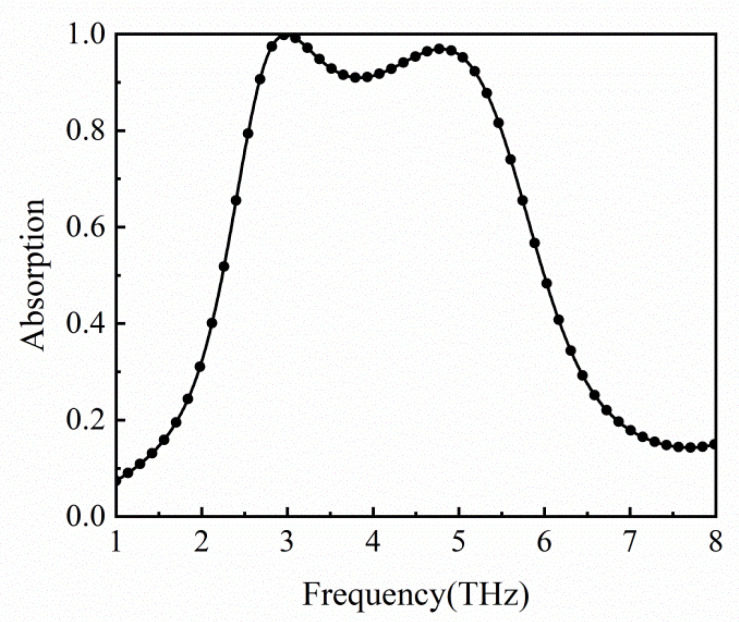
Absorption spectrum of the absorber for Fermi level *E_f_* = 0.8 eV. The bandwidth with absorption above 90% is 2.597 THz. There exist two absorption peaks, 2.995 THz and 4.808 THz.

The absorption spectrum is presented in Figure 3. The full-wave simulation was performed by CST Microwave Studio based on the finite element method (FEM). Adaptive tetrahedral mesh refinement has been used. In the simulation, the Fermi level of graphene is 0.8 eV, and the scattering time is assumed as 0.1 ps [42]. As can be seen from the figure, the bandwidth with absorption above 90% is 2.597 THz at the center frequency of 3.970 THz. There exist two absorption peaks, corresponding to absorption of 99.859% at 2.995 THz and 96.926% at 4.808 THz.

**Figure 4:**
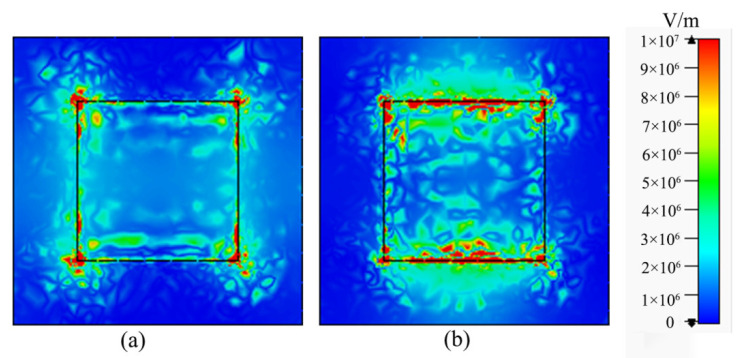
Electric field distributions of the graphene composite layer at resonance frequencies of (**a**) 2.995 THz and (**b**) 4.808 THz.

To grasp the physical mechanism of the absorber, the electric field distributions of the graphene composite layer at 2.995 THz and 4.808 THz are calculated, as shown in Figure 4. It is evident from Figure 4a that the electric field energy is mainly concentrated on the left and right edges of the graphene. The resonance direction of the dipole is horizontal, which is the transverse surface plasmon resonance mode. Similarly, in Figure 4b, the absorption peak at 4.808 THz results from the longitudinal plasmon mode. Thus, the strong absorption is attributed to the plasmon resonance, which results in the electric field energy being localized at the edge of the graphene. The reason for the wide bandwidth is the superposition of the longitudinal and transverse surface plasmon resonance modes [37].

**Figure 5:**
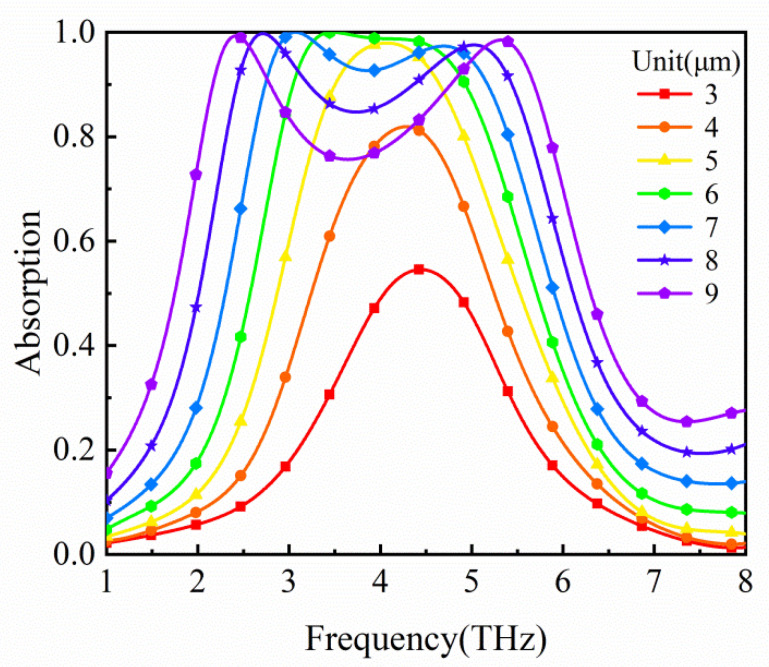
Absorption spectra of the absorber for different graphene side lengths.

According to the above analysis, the absorbing performance of the absorber is closely related to the graphene composite layer. So, we compute the influence of the graphene geometries on the absorption spectra, as shown in Figure 5. With the increase in the side length *L*, the bandwidth of the absorber broadens monotonously and the maximum absorptivity increases gradually. That is because the bigger the side length *L* is, the farther the spacing between two resonance frequencies of the transverse and longitudinal plasmon resonances is. Meanwhile, the ability to localize the electric field becomes stronger as the side length *L* increases.

**Figure 6:**
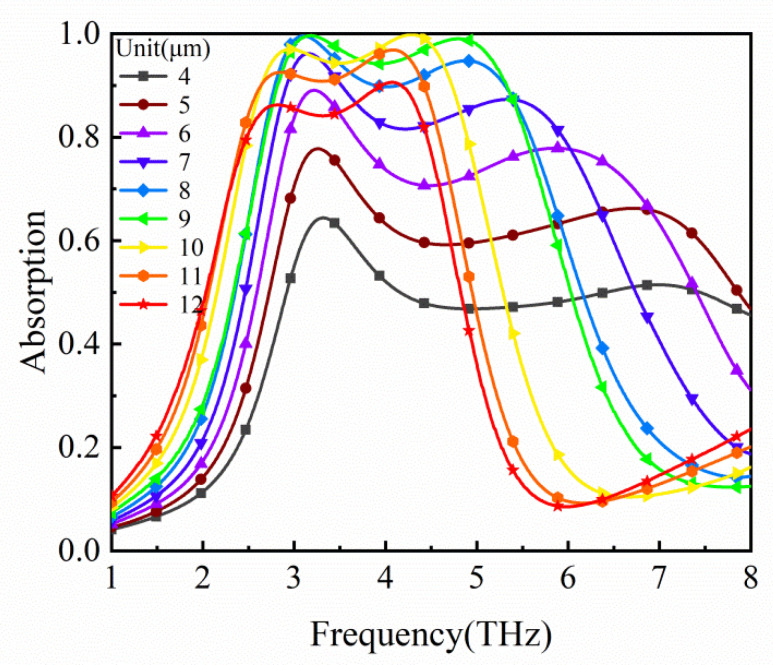
Absorption spectra of the absorber for different dielectric layer thicknesses.

In a perfect absorber design, the impedance matching plays an important role, which mainly depends on the thickness h_2_ of the dielectric layer for the proposed absorber. So, we calculated the influence of the thickness on the absorption spectra, as shown in Figure 6. It shows that with the increase in h_2_, the absorption firstly increases and then decreases, due to the realization of a good impedance match between the absorber and the free space near *h*_2_ = 9 μm. When *h*_2_ deviates from 9 μm, the impedance mismatch results in a decrease in absorption.

### 3.2. Frequency Tunability and Insensitivity

**Figure 7:**
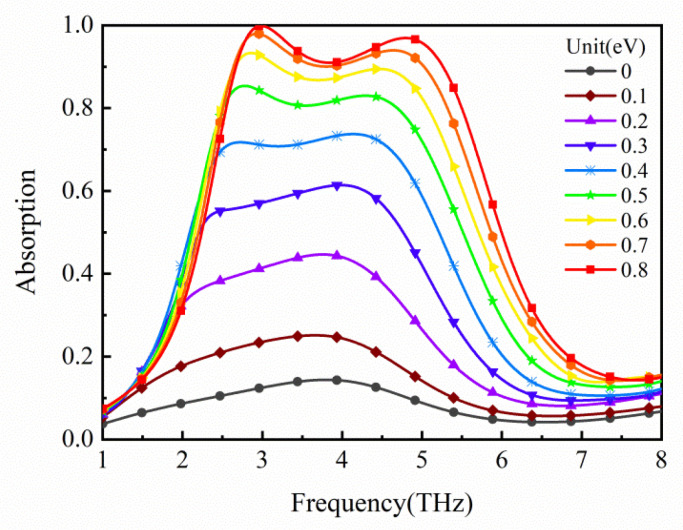
Absorption spectra of the proposed absorber with different Fermi levels *E_f_* of graphene (0–0.8 eV).

**Figure 8:**
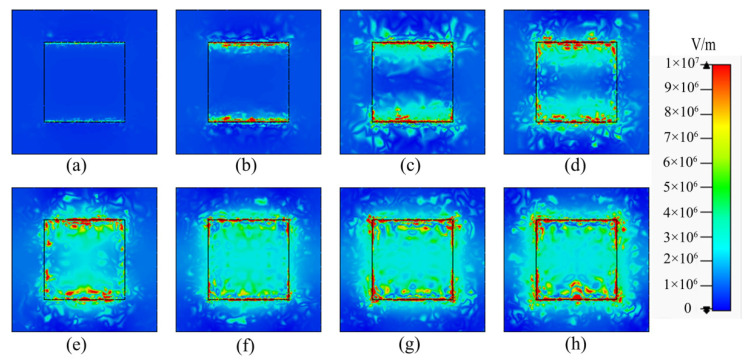
Electric field distributions of the graphene composite layer at the center frequency of 3.970 THz for different Fermi levels of 0–0.7 eV (**a**–**h**).

The tunability of the absorber is realized by graphene, because the Fermi level of the graphene can be adjusted by changing the applied voltage. Graphene in a floating gate can capture the tunneled electrons under the positive applied voltage, which increases the charge density. On the contrary, graphene, under the reverse applied voltage, can release electrons, leading to a decrease in its charge density. The relationship between Fermi level *E_f_* and charge density $n_{g}$ of graphene is as follows [34,43]: $E_{f}\approx \mu_{g}=\mathrm{sgn}\left(n_{g}\right)\hbar v_{F}\sqrt{\left(\pi \left|n_{g}\right|\right)}$, where $v_{F}=0.9\times 10^{6}\;m\cdot s^{-1}$ is the Fermi velocity. The change of charge density is the reason why graphene has tunability. To confirm the tunable capacity of the absorber, we calculated its absorption spectra for different *E_f_* in Figure 7. What can be clearly seen in this figure is the continual growth of the maximum absorption from 14.405% to 99.864% when varying the *E_f_* from 0 to 0.8 eV. When the Fermi level increases to 0.5 eV, the absorptivity is over 80%. Furthermore, the electric field distributions of the graphene composite layer at the center frequency of 3.970 THz for different Fermi levels are shown in Figure 8. It can be found that the resonance is enhanced due to the increase in surface plasmon at the upper and lower edges in Figure 8a–h. When *E_f_* ≥ 0.5 eV, the increase in the absorption slows down and the electric field has a slight change. Moreover, as the *E_f_* changes from 0 eV to 0.7 eV, the electric field energy is continuously concentrated. The reason is that the higher the *E_f_*, the lower the intrinsic loss of graphene [29]. It is helpful for the enhancement of surface plasmon resonance and the improvement of absorbing ability.

**Figure 9:**
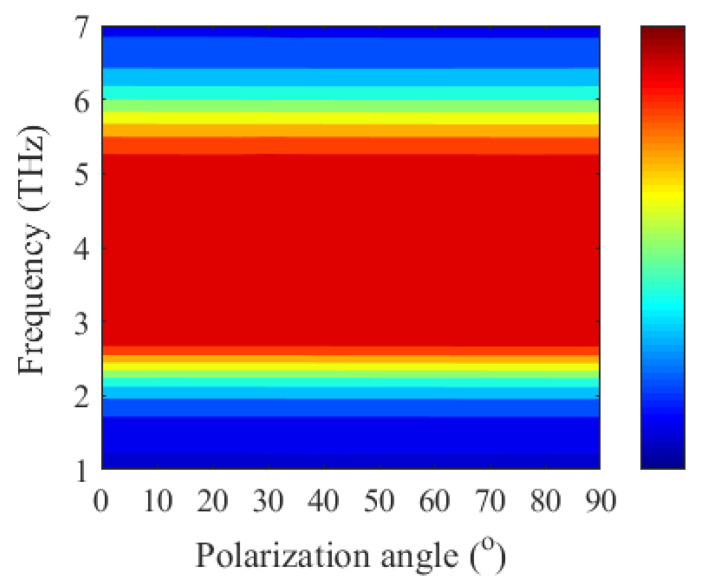
Absorption spectra of the absorber at various polarization angles.

**Figure 10:**
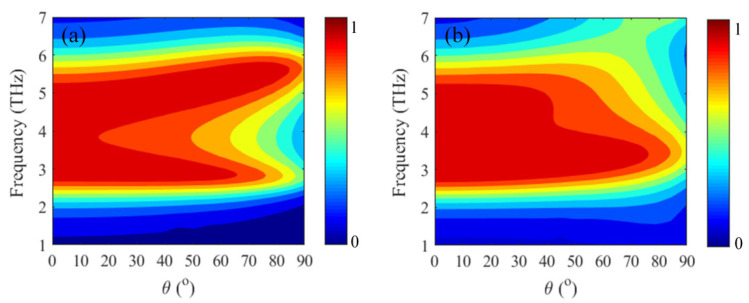
Absorption spectra with various incident angles for (**a**) transverse electric (TE) polarization and (**b**) transverse magnetic (TM) polarization.

In addition, we also investigate the sensitivity to polarization and the robustness of oblique incidence for the proposed absorber, and its corresponding absorption spectra are shown in Figure 9 and Figure 10. As can be seen from Figure 9, the absorber is polarization insensitive owing to the rotational symmetry of the periodic unit. One can find from Figure 10 that broadband strong absorption (absorption > 80%) can still be achieved for transverse electric (TE) and transverse magnetic (TM) polarization, when the incident angle is less than 45°.

## 4. Conclusions

In this paper, we propose a broadband, tunable, non-volatile, polarization-insensitive and wide-angle THz MMA based on a graphene floating gate. The absorption of the absorber can be dynamically tuned by changing the Fermi level of graphene and the depth of the tuning reaches up to 85.575%. More importantly, it can maintain a stable absorption performance even if the external bias voltage is disconnected. The proposed absorber shows potential applications in terahertz detecting, imaging, sensing and stealth areas.
